# Detection of Oxytetracycline Residuein Infant Formulaby High-Performance Liquid Chromatography)HPLC(

**Published:** 2011

**Authors:** Roya Khosrokhavar, Mir-Jamal Hosseini, Shahram Shoeibi, Behrooz Jannat

**Affiliations:** a *Food and Drug Laboratory Research center, Ministry of Health and Medical Education, Tehran, Iran.*; b *Food and Drug Control Laboratories, Ministry of Health and Medical Education, Tehran, Iran.*; c *Departments of Toxicology and Pharmacology, School of Pharmacy, Zanjan University of Medical Sciences, Zanjan, Iran.*; d *Departmants of Toxicology, School of Pharmacy, Shahid Beheshti University of Medical Sciences, Tehran, Iran.*

**Keywords:** Oxytetracycline, Infant formula, High-performance liquid chromatography, Ultraviolet detection

## Abstract

Determination of drug residues in food is of great importance due to their toxicity. Long-term exposure with low level of drug residues could be important, especially in children. Based on document study, oxytetracycline )OTC) is a prophylaxis and treatment agent for great number of diseases and possesses a broad spectrum activity against many pathogenic organisms and can be toxic or cause allergic reactions in some hypersensitive individual’s if the residues of drug exist in the infant formula. The previous studies show that using high-performance liquid chromatography (HPLC) is useful for OTC detection in milk. Therefore, we decided to measure OTC in infant formula. The purpose of this study was to investigate residual OTC in consuming infant formula using HPLC. The limit of quantification (LOQ) was 51 ng/mL. This result showed that the samples had no residues of OTC in infant formula from different companies.

## Introduction

“Infant formula as a breast-milk substitute is specially manufactured to satisfy, by itself, the nutritional requirement of infants during the first months of life up to the introduction of appropriate complementary feeding” (1). Infant formulas are of two basic types: milk-based and milk-free. Each type is available as concentrated liquid, ready-to-feed form and powdered varieties (2). The occurrence of antibiotic residues in infant formulas (milk-based) arising from its veterinary use is a case of concern to parents, pediatricians, nutritionists and particularly food control organizations worldwide, because of possible toxic or allergic reactions and the possibility that pathogenic organisms can be resistant to the antibiotics. It must be noticed that infants as target group (formula consumers) are more sensitive to above mentioned effects, so, these products should not contain contaminants or undesirable substances in amount which may represent a hazard to health of the infant (1, 3).

Tetracyclines (TC_S_) are the most recurrent prescribed antibiotics since their discovery in the mid-1900s. They have played an important role in sustaining health among the physicians and veterinarians. The therapeutics has been of particular interest for their use in food producing animals because of their broad spectrum activity and low cost. They (TC_S_) are used for preventing and controlling diseases and also as feed additives to promote the weight gain and to increase thefeed conversion efficiency and the amount of produced milk as well. TC_S_ are approved to be used in food producing animals with tolerances ranging from 0.7 to 2 PPM in commonly consumed animal tissue and products. Tolerances for less commonly consumed tissue reach 12 PPM (4, 5). TC_S_ can be successfully determined in various biological matrices, using high-performance liquid chromatography (HPLC) in the reverse-phase mode, with different detection modes such as spectrophotometry, fluorescence and mass spectrometry. The ultraviolet (UV) detection has low sensitivity, while mass spectrometry still requires costly instruments (3, 6, 7, 8). In Iran, some studies have been carried out on the presence of tetracyclines in food and infant formula.

The present study was conducted to describe the development in validation of an analytical method in order to detect and determine the Oxytetracycline (OTC) residue in commercially-available infant formula received in Food and Drug Control Laboratories 2008-2009.

## Experimental

A total of 30 samples of commercial infant formula were bought from supermarkets in Tehran for OTC hydrochloride analysis by HPLC. OTC hydrochloride of high purity (> 99%) and HPLC grade solvents were obtained from sigma (sigma, USA). A standard stock solution of OTC was prepared in methyl alcohol and stored at -20°C. Working standard solutions were daily prepared. All solutions had to be wrapped in aluminum foil since OTC is unstable and often changes from yellow to brown with exposure to light.

The chromatographic analysis was carried-out in a Dionex high-performance liquid chromatograph (München, Germany and Sunnyvale, CA, USA) equipped with a Dionex P680 Pump and a Dionex Rodyne Valre Injector. The analytical column which operates at room temperature was ACE C18-A3681, 250×4.6 mm ID from Dionex, and the analysis involving UV detection was performed in a Dionex UVD 170U/340U UV/Vis absorbance detector (The wavelength of the UV-Visible detector was set at 355 nm). The Chromeleon (version 6.60, Dionex) software was used to control the system. At the stage of extraction, Boatto *et al.* and Hosseini M *et al.* method has been used (9, 7).

Then the sample was filtered through a 0.45 µm syringe filter (Millipore) and 50 µL of the solution was injected to the HPLC. Calibration curve was constructed in five spiked concentrations of OTC (0.1, 0.2, 0.5, 0.7, 1 µg/mL) and then, samples were injected into the HPLC system and peak area of OTC was plotted versus OTC concentration. Later, the linearity of the method was evaluated. Linear least squares regression analysis gave a correlation coefficient of r^2^ = 0.995 and a calibration curve of (Y = 1.0946(x) + 0.0424) was concluded (Y = peak area of OTC and X = amount of OTC in µg/mL). The results were expressed as mean ± RSD of five samples by using Student’s t-test for data and then, determining the coefficient of variation (CV) for each concentration.

## Results and Discussion

The average recoveries of OTC from milk samples were suitable (more than 75%) at three different concentrations (0.1- 1 µg/mL) (Tables 1 and 2). Results of precision and accuracy were in acceptable range. The percentage of correlation variation was lower than 6% .The limit of detection (LOD) and limit of quantification (LOQ) were 35 and 51 ng/mL, respectively. Method specificity evaluated with endogenous interferences by extracting and analyzing blank samples from different sources. The chromatograms recorded at 355 nm were free of interfering extraneous peak, in the extracts of blank as well as in spiked samples. The retention time of target compound was 6.6 min.

**Table 1:** Recovery of oxytetracycline from milk Data are shown in average form (n = 5); coefficients of variation in parentheses (%).

**Added OTC (µg/mL)**	**Recovery% (µg/mL)**	
	**Inter-day**	**Intra-day**
0.1	77.2%	75.1%
0.5	79.6%	77.8%
1	85.9%	82.2%

**Table 2:** Intra and Inter-calibration analysis of oxytetracycline (n = 5) in spiked samples

**Added OTC (µg/mL)**	**RSD% (µg/mL)**	
	**Inter-day**	**Intra-day**
0.1	5.3	5.9
0.5	4.3	5.1
1	2.7	3.8

Tetracyclines (TC_S_) are widely distributed and potentially toxic elements in children, especially infants and young children which are at the pick of growth. Previous studies have shown toxic effect of OTC including allergic reaction (immunosuppressive effects), microbial resistant, liver toxicity and photo-toxicity (6, 8). In addition, Oxytetracycline (OTC) located in this family with a broad-spectrum bacteriostatic activity and applied in animal husbandry, is used to prevent the treatment of bacterial infections and increase growth rates .It could inhibited protein synthesis and cell growth of sensitive bacteria (8).

In response to protecting humans from the exposure to these drug residues in milk or infant formula, several European countries have established maximum residues limits (MRLs) for OTC in milk (0.1 µg/mL) and no drug residues in infant formula (1, 10, 11). In literature review, both microbiological and chromatographical methods have been described for monitoring tetracyclines in milk, infant formula and animal tissues and proposed that HPLC is the best method for OTC determination in liquid samples. Results of our study showed that there are no residues of OTC in infant formula samples. Although, OTC residue in infant formula samples were not detectable, fairly low cost and broad spectrum activity of OTC indicates the need for the awareness of the toxic effect of this drug and other drugs. In addition, it is suitable that total average of TC_S_ in milk and infant formula was evaluated.

In our study, the HPLC-UV detector allowed the separation and identification of OTC with a suitable retention time (6.6 min) without any interfering extraneous peak. The results show that HPLC techniques have provided accurate and reliable assay of OTC in infant formula. It seems that this method would be useful for routine monitoring of OTC residues in infant formula. It is necessary to have a comprehensive survey to determine the amount of drugs in products and indeed some rules are needed to be executed in this regard to minimize the contamination to the least possible amount and to preclude its adverse effect. Therefore, due to the importance of baby food and infant formula, more researches are to be conducted for designing quality improvement and safety of food stuff such as milk, infant formula and animal tissues from any drug residues.
